# Propyl 3-oxo-2,3-dihydro-1,2-benzothia­zole-2-carboxyl­ate

**DOI:** 10.1107/S1600536811028613

**Published:** 2011-08-02

**Authors:** Xiang-hui Wang, Jian-xin Yang, Cheng-hang You, Qiang Lin

**Affiliations:** a Institute of Environmental Science and Engineering, Kunming University of Science and Technology, Kunming 650093, People’s Republic of China; b Hainan University Materials and Chemical Engineering, Haikou 570228, People’s Republic of China; c Hainan Provincial Fine Chemical Engineering Center, Hainan University, Haikou 570228, People’s Republic of China; d College of Chemistry and Chemical Engineering, Hainan Normal University, Haikou 571100, People’s Republic of China

## Abstract

The title compound, C_11_H_11_NO_3_S, was synthesized by the reaction of benzo[*d*]isothia­zol-3(2*H*)-one with propyl carbono­chloridate in toluene. The benzoisothiazolone ring system is approximately planar with a maximum deviation from the mean plane of 0.0226 (14) Å for the N atom. Weak inter­molecular C—H⋯O hydrogen bonding occurs in the crystal structure.

## Related literature

For background to the synthesis of benzoisothiazolone derivatives, see: Davis (1972 ▶); Elgazwy & Abdel-Sattar (2003 ▶). For their biological activity, see: Taubert *et al.* (2002 ▶). For related structures, see: Xu *et al.* (2005 ▶, 2006 ▶); Cavalca *et al.* (1969 ▶, 1970 ▶).
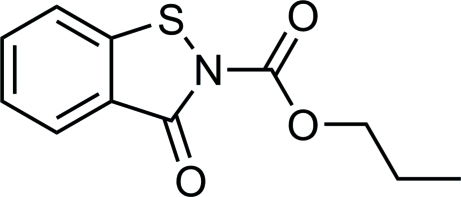


## Experimental

### 

#### Crystal data

C_11_H_11_NO_3_S
*M* *_r_* = 237.27
Monoclinic, 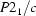

*a* = 16.235 (7) Å
*b* = 5.123 (2) Å
*c* = 12.791 (6) Å
β = 90.720 (7)°
*V* = 1063.7 (8) Å^3^
*Z* = 4
Mo *K*α radiation
μ = 0.29 mm^−1^
*T* = 153 K
0.35 × 0.25 × 0.20 mm

#### Data collection

Rigaku AFC10/Saturn724+ diffractometer
Absorption correction: multi-scan (*ABSCOR*; Higashi, 1995 ▶) *T*_min_ = 0.904, *T*_max_ = 0.943
8491 measured reflections
2766 independent reflections
2224 reflections with *I* > 2σ(*I*)
*R*_int_ = 0.031

#### Refinement

*R*[*F* ^2^ > 2σ(*F* ^2^)] = 0.039
*wR*(*F* ^2^) = 0.099
*S* = 1.00
2766 reflections
146 parameters
H-atom parameters constrained
Δρ_max_ = 0.35 e Å^−3^
Δρ_min_ = −0.25 e Å^−3^

### 

Data collection: *CrystalClear* (Rigaku, 2008 ▶); cell refinement: *CrystalClear*; data reduction: *CrystalClear*; program(s) used to solve structure: *SHELXS97* (Sheldrick, 2008 ▶); program(s) used to refine structure: *SHELXL97* (Sheldrick, 2008 ▶); molecular graphics: *SHELXTL* (Sheldrick, 2008 ▶); software used to prepare material for publication: *SHELXTL* and *publCIF* (Westrip, 2010 ▶).

## Supplementary Material

Crystal structure: contains datablock(s) I, global. DOI: 10.1107/S1600536811028613/fl2349sup1.cif

Structure factors: contains datablock(s) I. DOI: 10.1107/S1600536811028613/fl2349Isup2.hkl

Supplementary material file. DOI: 10.1107/S1600536811028613/fl2349Isup3.cml

Additional supplementary materials:  crystallographic information; 3D view; checkCIF report

## Figures and Tables

**Table 1:** Hydrogen-bond geometry (Å, °)

*D*—H⋯*A*	*D*—H	H⋯*A*	*D*⋯*A*	*D*—H⋯*A*
C2—H2⋯O2^i^	0.95	2.60	3.437 (3)	148

Symmetry code: (i) 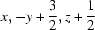.

**Fig. 1.**
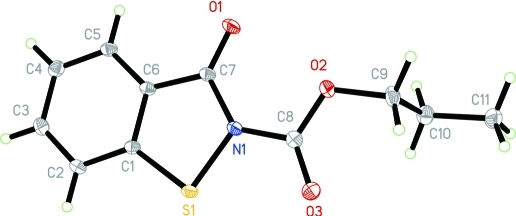
Title molecule showing the 30% probability displacement ellipsoids and the atomnumbering scheme.

**Fig. 2.**
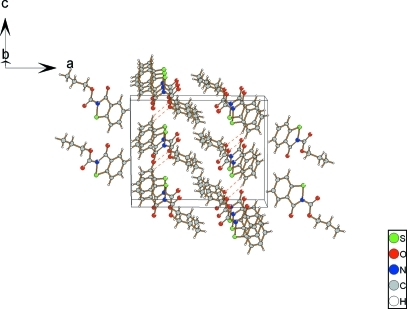
The crystal packing of the title compound, showing a hydrogen-bonded (dashed lines) molecular chain.

## supplementary crystallographic information

### Comment

1,2-benzisothiazol-3(2*H*)-ones are a class of compounds with a wide spectrum of biological activities (Davis, 1972), Elgazwy & Abdel-Sattar, 2003). 1, 2-Benzisothiazolone derivatives have been reported to possess high antibacterial and antifungal activity (Taubert *et al.*, 2002). In view of the importance of the 1,2-benzisothiazol-3(2*H*)-ones, the title compound, (I), was synthesized and characterized by X-ray diffraction.

The molecular structure of the title compound is shown in Fig. 1. In the molecule, the benzisothiazolone ring system is approximately planar with a maximum deviation from the mean plane of 0.0226 (14) A ° for the N atom, and the C8—O2—C9—C10 torsion angle is 85.16 (18)°. Weak intermolecular C—H···O hydrogen bonding occurs in the crystal structure (Table 1, Fig. 22))..

### Experimental

A toluol solution (20 ml) containing benzo[*d*]isothiazol-3(2*H*)-one (1.51 g, 0.01 mol) was added dropwise to a solution of propyl carbonochloridate (1.22 g, 0.01 mol) in toluol (20 ml) under stirring on an ice-water bath. The reaction mixture was stirred at room temperature for 4.5 h to afford the title compound (1.55 g, yield 65.5%). Single crystals suitable for X-ray measurements were obtained by recrystallization of the title compound from cyclohexane at room temperature.

### Refinement

The H atoms were placed at calculated positions and refined in riding mode, with the carrier atom-H distances = 0.95 Å for aryl, 0.99 for methylene, 0.98 Å for the methyl. The *U*iso values were constrained to be 1.5*U*eq of the carrier atom for the methyl H atoms and 1.2*U*eq for the remaining H atoms.

### Crystal data

**Table:**

C_11_H_11_NO_3_S	*F*(000) = 496
*M**_r_* = 237.27	*D*_x_ = 1.482 Mg m^−^^3^
Monoclinic, *P*2_1_/*c*	Mo *K*α radiation, λ = 0.71073 Å
*a* = 16.235 (7) Å	Cell parameters from 3253 reflections
*b* = 5.123 (2) Å	θ = 3.0–29.1°
*c* = 12.791 (6) Å	µ = 0.29 mm^−^^1^
β = 90.720 (7)°	*T* = 153 K
*V* = 1063.7 (8) Å^3^	Block, pink
*Z* = 4	0.35 × 0.25 × 0.20 mm

### Data collection

**Table:**

Rigaku AFC10/Saturn724+ diffractometer	2766 independent reflections
Radiation source: Rotating Anode	2224 reflections with *I* > 2σ(*I*)
graphite	*R*_int_ = 0.031
Detector resolution: 28.5714 pixels mm^-1^	θ_max_ = 29.1°, θ_min_ = 3.2°
phi and ω scans	*h* = −20→22
Absorption correction: multi-scan (*ABSCOR*; Higashi, 1995)	*k* = −6→6
*T*_min_ = 0.904, *T*_max_ = 0.943	*l* = −16→17
8491 measured reflections	

### Refinement

**Table:**

Refinement on *F*^2^	Primary atom site location: structure-invariant direct methods
Least-squares matrix: full	Secondary atom site location: difference Fourier map
*R*[*F*^2^ > 2σ(*F*^2^)] = 0.039	Hydrogen site location: inferred from neighbouring sites
*wR*(*F*^2^) = 0.099	H-atom parameters constrained
*S* = 1.00	*w* = 1/[σ^2^(*F*_o_^2^) + (0.0506*P*)^2^ + 0.316*P*] where *P* = (*F*_o_^2^ + 2*F*_c_^2^)/3
2766 reflections	(Δ/σ)_max_ < 0.001
146 parameters	Δρ_max_ = 0.35 e Å^−^^3^
0 restraints	Δρ_min_ = −0.25 e Å^−^^3^

### Special details

**Table:**

Geometry. All e.s.d.'s (except the e.s.d. in the dihedral angle between two l.s. planes) are estimated using the full covariance matrix. The cell e.s.d.'s are taken into account individually in the estimation of e.s.d.'s in distances, angles and torsion angles; correlations between e.s.d.'s in cell parameters are only used when they are defined by crystal symmetry. An approximate (isotropic) treatment of cell e.s.d.'s is used for estimating e.s.d.'s involving l.s. planes.
Refinement. Refinement of *F*^2^ against ALL reflections. The weighted *R*-factor *wR* and goodness of fit *S* are based on *F*^2^, conventional *R*-factors *R* are based on *F*, with *F* set to zero for negative *F*^2^. The threshold expression of *F*^2^ > σ(*F*^2^) is used only for calculating *R*-factors(gt) *etc*. and is not relevant to the choice of reflections for refinement. *R*-factors based on *F*^2^ are statistically about twice as large as those based on *F*, and *R*- factors based on ALL data will be even larger.

### Fractional atomic coordinates and isotropic or equivalent isotropic displacement parameters (Å^2^)

**Table:**

	*x*	*y*	*z*	*U*_iso_*/*U*_eq_	
S1	0.25453 (2)	0.70918 (8)	0.71855 (3)	0.02263 (12)	
O1	0.16642 (7)	0.7439 (2)	0.43840 (8)	0.0285 (3)	
O2	0.28813 (7)	1.0985 (2)	0.46620 (9)	0.0289 (3)	
O3	0.34796 (8)	1.1023 (3)	0.62778 (10)	0.0339 (3)	
N1	0.24529 (8)	0.8149 (3)	0.59025 (10)	0.0210 (3)	
C1	0.17595 (9)	0.4842 (3)	0.69666 (11)	0.0195 (3)	
C2	0.14578 (10)	0.3037 (3)	0.76888 (12)	0.0227 (3)	
H2	0.1671	0.2966	0.8383	0.027*	
C3	0.08395 (10)	0.1360 (3)	0.73593 (13)	0.0256 (3)	
H3	0.0628	0.0116	0.7837	0.031*	
C4	0.05157 (10)	0.1449 (3)	0.63348 (13)	0.0258 (3)	
H4	0.0088	0.0285	0.6130	0.031*	
C5	0.08200 (9)	0.3230 (3)	0.56251 (12)	0.0226 (3)	
H5	0.0608	0.3292	0.4930	0.027*	
C6	0.14445 (9)	0.4939 (3)	0.59480 (11)	0.0189 (3)	
C7	0.18296 (9)	0.6918 (3)	0.52901 (12)	0.0203 (3)	
C8	0.29898 (9)	1.0187 (3)	0.56431 (12)	0.0231 (3)	
C9	0.34321 (10)	1.3087 (3)	0.43295 (15)	0.0304 (4)	
H9A	0.3533	1.4290	0.4923	0.036*	
H9B	0.3163	1.4093	0.3760	0.036*	
C10	0.42408 (10)	1.2039 (4)	0.39541 (14)	0.0295 (4)	
H10A	0.4139	1.0737	0.3395	0.035*	
H10B	0.4532	1.1151	0.4539	0.035*	
C11	0.47784 (11)	1.4220 (4)	0.35365 (15)	0.0357 (4)	
H11A	0.4520	1.4964	0.2907	0.043*	
H11B	0.5322	1.3520	0.3364	0.043*	
H11C	0.4840	1.5583	0.4070	0.043*	

### Atomic displacement parameters (Å^2^)

**Table:**

	*U*^11^	*U*^22^	*U*^33^	*U*^12^	*U*^13^	*U*^23^
S1	0.0270 (2)	0.0248 (2)	0.01593 (19)	−0.00360 (15)	−0.00484 (14)	0.00027 (15)
O1	0.0359 (7)	0.0350 (7)	0.0145 (5)	−0.0040 (5)	−0.0035 (5)	0.0026 (5)
O2	0.0261 (6)	0.0344 (7)	0.0262 (6)	−0.0044 (5)	−0.0009 (5)	0.0112 (5)
O3	0.0381 (7)	0.0357 (7)	0.0279 (6)	−0.0132 (6)	−0.0037 (5)	−0.0001 (5)
N1	0.0242 (6)	0.0236 (7)	0.0151 (6)	−0.0010 (5)	−0.0010 (5)	0.0009 (5)
C1	0.0213 (7)	0.0193 (7)	0.0178 (7)	0.0021 (5)	−0.0014 (5)	−0.0029 (6)
C2	0.0276 (8)	0.0237 (8)	0.0166 (7)	0.0024 (6)	−0.0013 (6)	0.0017 (6)
C3	0.0299 (8)	0.0225 (8)	0.0245 (8)	0.0000 (6)	0.0042 (6)	0.0010 (6)
C4	0.0248 (8)	0.0254 (8)	0.0270 (8)	−0.0027 (6)	0.0001 (6)	−0.0052 (7)
C5	0.0232 (7)	0.0258 (8)	0.0188 (7)	0.0026 (6)	−0.0025 (6)	−0.0051 (6)
C6	0.0218 (7)	0.0199 (7)	0.0150 (7)	0.0044 (6)	−0.0006 (5)	−0.0030 (6)
C7	0.0226 (7)	0.0227 (8)	0.0156 (7)	0.0024 (6)	−0.0006 (6)	−0.0030 (6)
C8	0.0242 (8)	0.0221 (8)	0.0228 (8)	0.0023 (6)	0.0018 (6)	−0.0005 (6)
C9	0.0274 (8)	0.0260 (9)	0.0378 (10)	0.0003 (7)	0.0037 (7)	0.0136 (7)
C10	0.0300 (9)	0.0278 (9)	0.0308 (9)	−0.0015 (7)	0.0049 (7)	−0.0023 (7)
C11	0.0334 (9)	0.0434 (11)	0.0305 (9)	−0.0095 (8)	0.0042 (7)	0.0008 (8)

### Geometric parameters (Å, °)

**Table:**

S1—N1	1.7328 (15)	C4—C5	1.383 (2)
S1—C1	1.7393 (17)	C4—H4	0.9500
O1—C7	1.2162 (19)	C5—C6	1.398 (2)
O2—C8	1.329 (2)	C5—H5	0.9500
O2—C9	1.466 (2)	C6—C7	1.463 (2)
O3—C8	1.208 (2)	C9—C10	1.503 (2)
N1—C8	1.403 (2)	C9—H9A	0.9900
N1—C7	1.4195 (19)	C9—H9B	0.9900
C1—C6	1.395 (2)	C10—C11	1.519 (3)
C1—C2	1.400 (2)	C10—H10A	0.9900
C2—C3	1.383 (2)	C10—H10B	0.9900
C2—H2	0.9500	C11—H11A	0.9800
C3—C4	1.407 (2)	C11—H11B	0.9800
C3—H3	0.9500	C11—H11C	0.9800
			
N1—S1—C1	90.00 (7)	O1—C7—C6	127.56 (14)
C8—O2—C9	115.18 (13)	N1—C7—C6	107.29 (12)
C8—N1—C7	129.86 (13)	O3—C8—O2	127.15 (15)
C8—N1—S1	114.20 (10)	O3—C8—N1	120.72 (15)
C7—N1—S1	115.87 (11)	O2—C8—N1	112.13 (13)
C6—C1—C2	120.86 (14)	O2—C9—C10	111.65 (14)
C6—C1—S1	112.73 (12)	O2—C9—H9A	109.3
C2—C1—S1	126.40 (12)	C10—C9—H9A	109.3
C3—C2—C1	117.89 (14)	O2—C9—H9B	109.3
C3—C2—H2	121.1	C10—C9—H9B	109.3
C1—C2—H2	121.1	H9A—C9—H9B	108.0
C2—C3—C4	121.68 (15)	C9—C10—C11	110.97 (16)
C2—C3—H3	119.2	C9—C10—H10A	109.4
C4—C3—H3	119.2	C11—C10—H10A	109.4
C5—C4—C3	120.04 (15)	C9—C10—H10B	109.4
C5—C4—H4	120.0	C11—C10—H10B	109.4
C3—C4—H4	120.0	H10A—C10—H10B	108.0
C4—C5—C6	118.88 (15)	C10—C11—H11A	109.5
C4—C5—H5	120.6	C10—C11—H11B	109.5
C6—C5—H5	120.6	H11A—C11—H11B	109.5
C1—C6—C5	120.65 (14)	C10—C11—H11C	109.5
C1—C6—C7	114.07 (13)	H11A—C11—H11C	109.5
C5—C6—C7	125.28 (14)	H11B—C11—H11C	109.5
O1—C7—N1	125.15 (15)		
			
C1—S1—N1—C8	178.88 (12)	S1—N1—C7—O1	178.47 (13)
C1—S1—N1—C7	1.45 (12)	C8—N1—C7—C6	−179.01 (14)
N1—S1—C1—C6	−0.32 (12)	S1—N1—C7—C6	−2.07 (16)
N1—S1—C1—C2	178.34 (14)	C1—C6—C7—O1	−178.77 (16)
C6—C1—C2—C3	0.0 (2)	C5—C6—C7—O1	1.8 (3)
S1—C1—C2—C3	−178.55 (12)	C1—C6—C7—N1	1.79 (18)
C1—C2—C3—C4	−0.3 (2)	C5—C6—C7—N1	−177.67 (14)
C2—C3—C4—C5	0.6 (2)	C9—O2—C8—O3	0.9 (2)
C3—C4—C5—C6	−0.6 (2)	C9—O2—C8—N1	−179.00 (13)
C2—C1—C6—C5	−0.1 (2)	C7—N1—C8—O3	178.88 (15)
S1—C1—C6—C5	178.67 (12)	S1—N1—C8—O3	1.9 (2)
C2—C1—C6—C7	−179.57 (14)	C7—N1—C8—O2	−1.2 (2)
S1—C1—C6—C7	−0.82 (17)	S1—N1—C8—O2	−178.19 (10)
C4—C5—C6—C1	0.4 (2)	C8—O2—C9—C10	85.16 (18)
C4—C5—C6—C7	179.80 (14)	O2—C9—C10—C11	175.72 (15)
C8—N1—C7—O1	1.5 (3)		

### Hydrogen-bond geometry (Å, °)

**Table:**

*D*—H···*A*	*D*—H	H···*A*	*D*···*A*	*D*—H···*A*
C2—H2···O2^i^	0.95	2.60	3.437 (3)	148

Symmetry codes: (i) *x*, −*y*+3/2, *z*+1/2.
